# Beneficial Effect of Water-Based Exercise Training on Exercise Capacity in COPD Patients—a Pilot Study

**DOI:** 10.3389/fresc.2021.728973

**Published:** 2021-11-17

**Authors:** Noppawan Charususin, Thiti Sricharoenchai, Karan Pongpanit, Kornanong Yuenyongchaiwat, Phuwarin Namdaeng, Jitanan Laosiripisan, Piyapa Keawutan

**Affiliations:** ^1^Department of Physical Therapy, Faculty of Allied Health Sciences, Thammasat University, Pathumthani, Thailand; ^2^Research Unit of Physical Therapy in Respiratory and Cardiovascular Systems, Thammasat University, Pathumthani, Thailand; ^3^Department of Internal Medicine, Faculty of Medicine, Thammasat University, Pathumthani, Thailand

**Keywords:** water-based exercise, chronic obstructive pulmonary disease, pulmonary rehabilitation, exercise capacity, inspiratory muscle strength

## Abstract

**Background:** Chronic obstructive pulmonary disease (COPD) is a common, preventable, and treatable condition, characterized by persistent airflow limitation. Exercise training is a core component of pulmonary rehabilitation in people with COPD. Water-based exercise has been studied, but it remains unclear whether water-based exercise program leads to the improvement in respiratory function, muscle strength, balance ability, and exercise capacity. We aim to study the effect of an 8-week water-based exercise program on respiratory function, muscle strength, balance ability, and exercise capacity in people with COPD.

**Methods:** Fourteen stable COPD participants (FEV_1_ 56.8 ± 24.6%pred) were recruited and randomized into a water-based exercise or a land-based exercise group. Both groups were trained for 8 weeks, two sessions per week. Pulmonary function, respiratory muscle strength, peripheral muscle strength, balance ability, exercise capacity [6-min walking test (6MWT), incremental shuttle walk test (ISWT), and endurance shuttle walk test (ESWT)] were assessed at baseline and at the end of the program. ANCOVA was used to conduct between-group comparisons of outcomes after adjusting for pre-intervention values.

**Results:** Baseline characteristics of participants were not significantly different between the two groups (*p* ≥ 0.05). After the 8-week training program, participants in the intervention group achieved larger gains in ESWT (Δ663.4 ± 279.5 vs. Δ45.4 ± 93.2 s, *p* = 0.001). In addition, maximal inspiratory pressure (MIP) was significantly increased more in the intervention group (Δ11.1 ± 7.8 vs. Δ1.1 ± 5.7 cmH_2_O, *p* = 0.026). However, no significant differences in pulmonary function, peripheral muscle strength, balance ability variables, 6MWD (*p* = 0.248), and ISWT (*p* = 0.506) were observed between the two groups.

**Conclusions:** The water-based exercise program could be recommended to the COPD rehabilitation program for improving the endurance exercise capacity and inspiratory muscle strength.

**Clinical Trial Registration:** www.thaiclinicaltrials.org, identifier: TCTR20210125005.

## Introduction

The main types of NCDs are cardiovascular diseases (heart attacks and stroke), cancers, diabetes, and chronic respiratory diseases (such as chronic obstructive pulmonary disease and asthma) are responsible for most deaths worldwide (1). Chronic respiratory diseases constitute a public health problem that impose a substantial burden. Especially, chronic obstructive pulmonary disease (COPD) is a major cause of chronic morbidity and mortality. COPD is a common, preventable and treatable disease that is characterized by persistent respiratory symptoms and airflow limitation that is due to airway and/or alveolar abnormalities usually caused by significant exposure to noxious particles or gases (1, 2). Dyspnea is the most notable exercise-limiting symptom of the disease, which leads to chronic avoidance of physical activities. As a result, low physical activity levels contribute to upper and lower limb muscle deconditioning and exercise capacity reduction, which impact negatively on health related quality of life (2, 3).

Pulmonary rehabilitation (PR) including exercise training, education, nutritional intervention, and psychosocial support is an integrated and standard care option for COPD patients (3, 4). It is the most effective non-pharmacological management to improve symptoms of dyspnea and fatigue, increase exercise capacity, improve health-related quality of life, and reduce hospital admissions (3–5). The most effective strategies for the rehabilitation of COPD is land-based exercise training. However, the general demographic of people with COPD referred to PR are of an older age, and a high proportion have physical comorbidity, such as musculoskeletal abnormalities, cerebrovascular disease, osteoarthritis, and obesity, which limit their ability to participate in land-based exercise training. There is a high drop-out rate to 66% from land-based exercise training. Therefore, performing land-based exercise training may considerably reduce the effectiveness and does not find significant differences when study in this training (6).

Even though water-based exercise has been studied (6–9), it remains unclear whether water-based exercise programs lead to an improvement in endurance exercise capacity, respiratory function, muscle strength, and balance ability. In a Cochrane review conducted in 2013, the authors concluded that there is limited quality evidence that water-based exercise training is safe and improves exercise capacity and quality of life in people with COPD immediately after training (7). Therefore, a comprehensive physical evaluation has been performed in this present study. We aimed to study the effect of an 8-week water-based exercise program on endurance exercise capacity, respiratory function, muscle strength, and balance ability in people with COPD. The second objective was to investigate the correlation between the change of primary outcome (endurance exercise capacity) and all secondary outcomes.

## Materials and Methods

### Study Design, Data Collection, and Randomization

Participants who had confirmed diagnosis of COPD according to the Global Initiative for Obstructive Lung Disease (GOLD) criteria, were in a stable phase age between 40 and 75 years old, and were referred for outpatient treatment at Thammasat university hospital were included in this study. The exclusion criteria were: Psychiatric disorders, cognitive disorders, progressive neurological or neuromuscular disorders, progressive musculoskeletal disorders, or severe orthopedic problems that impact in activity in daily life.

The participants gave written informed consent, answered the questionnaire, and gave personal data. This study was a randomized control trial with assessor blind (assessor and therapist would be different) due to the exercise interventions, it was not possible to blind the participants or therapist. Group allocation was performed by simple randomization using sealed opaque envelopes in random block sizes of four and six (order unknown to investigators) (10). They were divided into two groups: water-based exercise training and land-based exercise training (control group).

This study was approved by the Ethical Review Sub-Committee Board for Human Research Involving Sciences, Thammasat University, No.3 (COA No.110/2561). The clinical trial has been registered in Thai Clinical Trials Registry (the TCTR identification number is TCTR20210125005).

### Measurement

Pulmonary function was assessed by spirometry using MicroLab MK8 (11). Maximal inspiratory pressure (MIP) and maximal expiratory pressure (MEP) were registered at the mouth by an electronic pressure transducer (MicroRPM Micromedical, UK) (12). Peripheral muscle strength was assessed using hand-held dynamometer for quadriceps strength and hand grip dynamometer for hand grip strength (13). Postural balance was assessed by five time sits to stand (FTSTS) test and time up and go (TUG) (14). Exercise capacity was assessed by 6-min walk test (6MWT), incremental shuttle walk test (ISWT), and endurance exercise capacity was assessed by the endurance shuttle walk test (ESWT) following the recommendations of European Respiratory Society/American Thoracic Society (15, 16). The primary outcome was ESWT. The secondary outcomes were pulmonary function, respiratory muscle strength, peripheral muscle strength, postural balance, and exercise capacity. Participants have been assessed at baseline and the end of the program.

### Intervention

Water-based exercise group was performed in a hydrotherapy pool with maximum of 12 participants per session under supervision, with the water temperature at 34 degree Celsius and the water level between the clavicle and xiphisternum for each participant. The exercise program was performed at moderate intensity, and before the exercise program participants were tested to find their blood pressure, respiratory rate, oxygen saturation, peak expiratory flow rate (PEFR), dyspnea and legs fatigue scale, according to modified borg scale for dyspnea, and perceived exertion at an intensity rating of three to five. Training intensity was measured three times during each exercise session. If the intensity reported was below three, participants were encouraged to increase their intensity or to add equipment to increase the resistance of the exercise program. For the land-based exercise group, at the beginning of the training course, the physiotherapist instructed participants about the correct exercise program as closely as with the water-based exercise group. Subjects in the land-based training group exercised on their own, but closely followed the instruction given at the beginning of the course and the exercise handbook. We followed up with the land-based training group by telephone call twice a week. Both training interventions were matched as closely as possible for training intensity, duration, muscles, and also the training progression. These protocols were modified from the study of McNamara et al. (8) (Table 1) and the training progression was adapted from Felcar et al. (17) (Table 2). Both groups were trained 60 min per session, 2 sessions per week for 8 weeks.

**Table 1:** Land-based and water-based exercise program (8).

**Exercise phase**	**Duration**	**Exercise program**
Warm up	7 min	•Breathing control •Straight leg kick (stretching of lower limbs) •Kicking & Punching •Jogging (stationary)
Lower limb exercise 1	10 min	•Straight leg kick forwards, sideways and backwards •High knee shuffle with opposite elbow to opposite high knee •Straight leg kick with arm reach to opposite toe •Straight leg walking
Resting	2 min	•Breathing control and hydration
Lower limb exercise 2	10 min	•Bunny hops •Single leg jumps •Star jumps •Seated leg cycling (stationary)
Resting	2 min	•Breathing control and hydration
Respiratory muscle stretches gymnastic	10 min	•Slowly breath in, gradually elevating both shoulders. After taking a deep breath, slowly breathe out and pulling back both shoulders. •Place both hands on upper chest. Pull back your elbows and pull your chest up while lifting your chin and deep breath in, slowly breath out through your mouth and relax. •Put your hands in front of your body at shoulder height. Slowly breathe in, move your hands frontward with lean trunk flexion, stretch your back, move your hand down and slowly breath out. •Hold a towel with both hands outstretched at shoulder height. After taking a deep breath, move your arms up while breathing out slowly. •Hold one hand behind your head. Take a deep breath. While slowly breathing out, raising elbow as high as is possible. Repeat on the opposite side.
Resting	2 min	•Breathing control and hydration
Upper limb exercise	10 min	•Raise your hands in front of your body at shoulder height and bring your hand down. •Extend both arms out straight to your side at shoulder height and put down on your side. •Raise your hands in front of you at shoulder height and reach out to the sides of your body with both hands. •Place your arms hanging by your sides, bending the hand toward the shoulder and return to starting position. Use dumbbell to perform the exercise program
Cool down	7 min	•Clasp and raise your hands over head, lateral bending your trunk to both sides. •Breathing control

**Table 2:** Progression of exercise program for land-based and water-based exercise group (12).

**Exercise phase**	**Exercise program**	**Intensity**
Lower limb exercise	•Straight leg kick forwards, sideways, and backwards •High knee shuffle with opposite elbow to opposite high knee •Straight leg kick with arm reach to opposite toe	Week 1^st^-2^nd^ : 10 reps Week 2^nd^-4^th^ : 15 reps Week 4^th^-6^th^ : 20 reps Week 6^th^-8^th^ : 25 reps
	•Straight leg walking	Week 1^st^-2^nd^ : 75% 6MWT/3* Week 2^nd^-4^th^ : 80% 6MWT/3* Week 4^th^-6^th^ : 85% 6MWT/3* Week 6^th^-8^th^ : 90% 6MWT/3*
	•Seated leg cycling (stationary)	Week 1^st^-2^nd^ : 1 min (Modified Borg scale 4–6) Week 2^nd^-4^th^ : 1 min (Modified Borg scale 4-6) Week 4^th^-6^th^ : 1 min (Modified Borg scale 4-6) Week 6^th^-8^th^ : 1 min (Modified Borg scale 4-6)
Upper limb exercise	•Raise your hands in front of your body at shoulder height and bring your hand down. •Extend both arms out straight to your side at shoulder height and put down on your side. •Raise your hands in front of you at shoulder height and reach out to the sides of your body with both hands. •Place your arms hanging by your sides, bending the hand toward the shoulder and return to starting position.	Week 1^st^-2^nd^ : 10 reps Week 2^nd^-4^th^ : 15 reps Week 4^th^-6^th^ : 20 reps Week 6^th^-8^th^ : 25 reps

* *average speed of the 6MWT was measured in meters per minute. This speed was used to calculate in steps per minute, which was used to dictate the “beats per minute” tempo of the metronome*.

Compliance with the training protocol was assessed by physiotherapist during the training sessions in the water-based exercise group and by telephone call in the land-based exercise group.

### Statistical Analysis

Statistical analysis was performed using SPSS Version 21 and G-Power version 3.1.9.2 was used to calculate the sample size based on our pilot study and the study of McNamara et al. (8). To detect a minimally clinically important difference between groups of 203 m in the ESWT distance, with a degree of certainty (statistical power) of 80% and a risk for a type 1 error (α) <5%, a total of 14 participants (7 per group) were required to complete the study. Baseline data was verified using the descriptive statistics. Analysis of covariance (ANCOVA) was used to conduct between-group comparisons of outcomes. Pearson correlation was used to analyze the correlation between outcomes. Statistical significance was set as *P* < 0.05.

## Results

**Figure 1:**
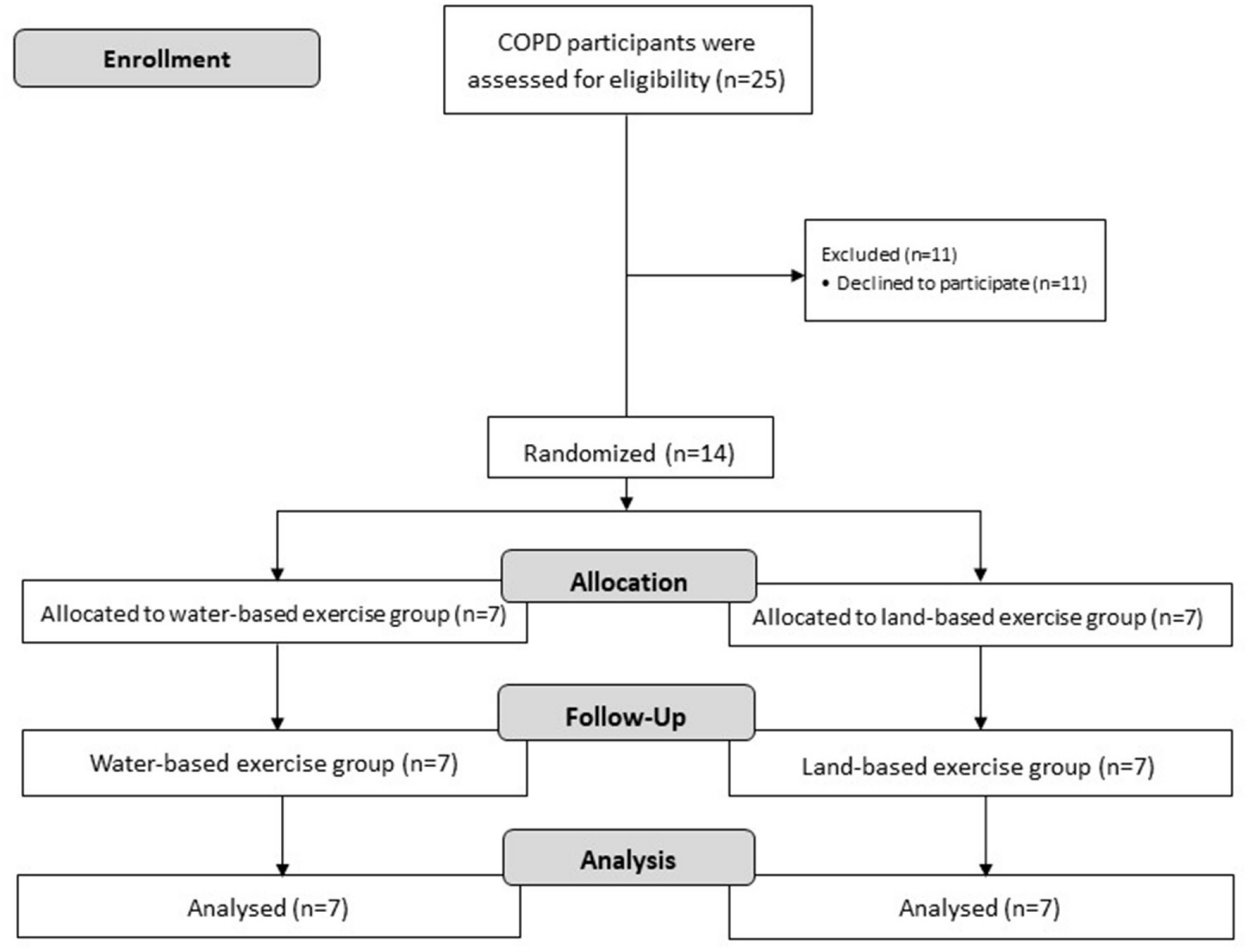
A diagram summarizing the flow of participants through the study.

Between January 2019 and October 2020, 25 patients were screened for study participation. A diagram summarizing the flow of participants through the study is presented in Figure 1. The most frequent reason for non-eligibility was declined to participate (*n* = 11). Baseline characteristics of included patients were not significantly different between the two groups (Table 3).

**Table 3:** Baseline characteristics.

**Variables**	**WBE group (***n*** = 7)**	**LBE group (***n*** = 7)**	***P*** **-value**
Age (year)	66 ± 8.1	69 ± 4.9	0.310
Gender (F/M)	2/5	2/5	-
Weight (kg)	65.2 ± 15.9	55.5 ± 13.5	0.243
Height (m)	159.7 ± 5.3	160.9 ± 3.5	0.641
BMI (kg/m^2^)	25.4 ± 4.5	21.4 ± 4.6	0.130
mMRC	1.7 ± 0.5	1.7 ± 0.9	1.000
FEV_1_ (%predicted)	51.2 ± 17.4	61.6 ± 30.1	0.472
FVC (%predicted)	60.2 ± 24.8	75.0 ± 23.2	0.289
FEV_1_/FVC (%)	58.7 ± 10.2	59.1 ± 11.0	0.516
MIP (cmH_2_O)	58.1 ± 25.0	67.0 ± 18.7	0.467
MEP (cmH_2_O)	98.6 ± 27.9	90.1 ± 36.2	0.634
Quadriceps strength (Nm)	128.1 ± 20.9	128.5 ± 23.5	0.553
Hand grip strength (Nm)	24.9 ± 9.0	22.1 ± 6.7	0.400
FTSTS (seconds)	13.9 ± 2.7	16.9 ± 7.4	0.345
TUG (seconds)	11.2 ± 1.3	16.4 ± 6.2	0.053
6MWT (meters)	335.0 ± 107.2	290.1 ± 96.0	0.426
ISWT (meters)	254.3 ± 132.1	185.7 ± 53.8	0.228
ESWT (seconds)	352.3 ± 245.5	570.0 ± 269.4	0.140

*Data are presented as mean ± SD*.

*WBE, water-based exercise; LBE, land-based exercise; BMI, body mass index; mMRC, modified Medical Research Council dyspnea scale; FEV_1_, forced expiratory volume in one second; FVC, forced vital capacity; %predicted, percentage of the predicted normal value; MIP, maximal inspiratory mouth pressure; MEP, maximal expiratory mouth pressure; FTSTS, five times sit to stand test; TUG, timed up and go test; 6MWT, six-minute walk test; ISWT, incremental shuttle walk test; ESWT, endurance shuttle walk test; ESWT*.

### Changes in Pulmonary Function, Respiratory and Peripheral Muscles Function, Balance Ability, and Functional Exercise Capacity Outcomes

Patients in the water-based exercise group exhibited significantly larger improvements in inspiratory (Δ11.1 ± 7.8 vs. Δ1.1 ± 5.7 cmH_2_O, *p* = 0.026), but not expiratory muscle strength in comparison to the land-based exercise group (*p* = 0.071) (Table 4). There were no statistically significant changes in pulmonary function and other muscle strength variables between the two groups. FTSTS and TUG were both improved within both groups after training with no statistically significant between-group differences. The water-based exercise group exhibited significantly larger improvements in ESWT (Δ663.4 ± 279.5 vs. Δ45.4 ± 93.2 s, *p* = 0.001), but not 6MWT and ISWT (*p* = 0.248 and *p* = 0.506, respectively). Moreover, there was a trend of reduction in dyspnea symptom after the ISWT and ESWT in both groups. Furthermore, ESWT was not significantly correlated with all pulmonary function, respiratory and peripheral muscles function, balance ability variables.

**Table 4:** Changes in pulmonary function, respiratory and peripheral muscles function, balance ability, and functional exercise capacity outcomes.

**Variables**	**WBE group (***n*** = 7)**		**LBE group (***n*** = 7)**		**Adjusted differences (95%) at post training**	***P*** **-value**
	**Pre-training**	**Post-training**	**Pre-training**	**Post-training**		
**Pulmonary function**						
FEV_1_ (%pred)	51.2 ± 17.4	53.0 ± 18.4	61.6 ± 30.1	65.7 ± 37.2	−2.6 (−26.1 to 20.9)	0.811
FVC (%pred)	60.2 ± 24.8	73.8 ± 24.6	75.0 ± 23.2	75.9 ± 25.3	6.3 (−22.7 to 35.2)	0.641
FEV_1_/FVC (%)	58.7 ± 10.2	57.9 ± 13.4	59.1 ± 11.0	58.4 ± 18.6	−15.8 (−32.7 to 1.2)	0.266
**Respiratory and peripheral muscles function**						
MIP (cmH_2_O)	58.1 ± 25.0	69.3 ± 24.8^†^	67.0 ± 18.7	68.1 ± 21.9	10.1 (1.5–18.7)	0.026*
MEP (cmH_2_O)	98.6 ± 27.9	131.0 ± 55.5^†^	90.14 ± 36.2	92.3 ± 30.9	29.1 (−2.9 to 61.2)	0.071
Quadriceps strength (Nm)	128.1 ± 20.9	132.8 ± 40.8	128.4 ± 23.5	124.5 ± 12.2	40.2 (14.8–65.5)	0.327
Hand grip strength (Nm)	24.9 ± 9.0	26.3 ± 8.0	22.1 ± 6.7	22.6 ± 6.7	1.5 (−2.4 to 5.4)	0.520
**Balance ability**						
FTSTS (sec)	13.9 ± 2.7	12.1 ± 2.2	16.9 ± 7.4	15.3 ± 5.4	−1.6 (−5.3 to 2.1)	0.368
TUG (sec)	11.2 ± 1.3	9.7 ± 1.5^†^	16.4 ± 6.2	14.4 ± 5.6^†^	−0.4 (−2.7 to 2.0)	0.741
**Functional exercise capacity**						
6MWT (m)	335.0 ± 107.2	360.6 ± 103.2	290.1 ± 96.1	292.0 ± 106.0	25.2 (−20.3 to 70.8)	0.248
ISWT (m)	254.3 ± 132.1	282.9 ± 120.2	185.7 ± 53.8.0	202.9 ± 78.9	16.7 (−36.8 to 70.3)	0.506
ESWT (sec)	352.3 ± 245.5	985.7 ± 304.7^†^	570.0 ± 269.4	615.4 ± 281.0	548.8 (274.5 to 823.1)	0.001*

*Data are presented as mean ± SD and mean (95% CI)*.

* *P-values are reported for between-group comparisons (analysis of covariance of post-training values adjusted for baseline values as covariates)*.

† *A statistically significant difference within groups (p < 0.05)*.

*WBE, water-based exercise; LBE, land-based exercise; FEV_1_, forced expiratory volume in one second; FVC, forced vital capacity; %pred, percentage of the predicted normal value; MIP, maximal inspiratory mouth pressure; MEP, maximal expiratory mouth pressure; FTSTS, five times sit to stand test; TUG, timed up and go test; 6MWT, six-minute walk test; ISWT, incremental shuttle walk test; ESWT, endurance shuttle walk test; ESWT*.

Both groups had a high and similar training adherence. The water-based exercise group completed 73.4 ± 17.2% of all training sessions while the land-based exercise group completed 71.7 ± 21.6% of all sessions. In addition, there was a trend of reduction in average dyspnea scale after each training sessions in both water-based exercise and land-based exercise group (-0.4 ± 0.2 vs.−0.2 ± 0.35, respectively).

## Discussion

This pilot study investigated the effects of water-based exercise on respiratory function, muscle strength, balance ability, and exercise capacity in people with chronic obstructive pulmonary disease. The outcome of the water-based exercise program has not been well-characterized and not widely reported. In this study, we address this relevant topic and provide the evidence showing the benefits of the water-based exercise on the COPD patients. We include a comprehensive physical evaluation of the patients and perform the randomized control trial to strengthen our study approaches. The main findings of this study are two profound improvements in endurance exercise capacity assessed by the ESWT (primary outcome) and in the inspiratory muscle strength in the COPD patients (*n* = 7) who engaged in the water-based exercise program for 8 weeks. Significant differences in pulmonary function, peripheral muscle strength, balance ability variables, the 6MWD, and the ISWT were not found between the two groups. Moreover, the correlations between the improvement in ESWT and the secondary outcomes were not found in this study.

First, the endurance exercise capacity which was measured by the ESWT was dramatically enhanced in favor of the water-based exercise group. This result could be explained by the buoyancy properties of water which supports the body weight, water resistance which increases exercise intensity, and the proposed effects of warm water (34 degrees Celsius) on muscle blood flow (18). These may allow the COPD patients to exercise at a higher intensity with a likelihood of dyspnea to be reduced. Felcar et al. previously found that patients with moderate-to-severe COPD who participated in a high-intensity exercise training in water generates results similar to our findings. After 6 months of training, significant improvements were seen in inspiratory, expiratory, and peripheral muscle strength; maximal and submaximal exercise capacity; quality of life and functional status (17). Moreover, other studies reported that the water-based exercise was more effective in improving endurance exercise capacity than the land-based exercise (6, 8).

Next, the participants in the intervention group achieved a larger gain in the inspiratory muscle strength, as indicated by a significant elevation of the MIP in the water-based exercise group compared to the control group (11 vs. 1 cmH_2_O) after training for 8 weeks. One of the explanations could be that the hydrostatic pressure placed on the chest wall while immersing in water, which adds load to the inspiratory muscles (8), may act as a training stimulus and may increase the work of breathing. Similar to the previous study, the water-based exercise can improve respiratory muscle strength and peripheral muscle strength in patients with COPD (19).

This pilot study encounters limitations which include the small sample size and the difference between the supervised and unsupervised training sessions between the two groups. Further studies should be conducted with a larger sample size under the same supervised training sessions between the water-based and land-based exercise groups. Different changes in the outcomes at the end of the program may be more pronounced in a study with a larger number of the patients. We noticed that the results related to balance and exercise capacity at the baseline showed better values in the water-based exercise group relative to the land-based exercise group. We also noted that the probability of these variables to be intensified at the end of the program is high in the water-based exercise group. Whether or not a high deviation in these variables underlies a non-significant difference between the two exercise groups at the baseline could be addressed by increasing the study sample size.

In conclusion, this pilot study shows the beneficial consequence of the water-based exercise program in COPD patients and suggests that this training program could potentially be incorporated to the COPD rehabilitation program to improve the endurance exercise capacity and inspiratory muscle strength. These findings could be further supported by studies that include a larger sample size under the same supervised training sessions between the water-based and land-based exercise groups.

## Data Availability Statement

The raw data supporting the conclusions of this article will be made available by the authors, without undue reservation.

## Ethics Statement

The studies involving human participants were reviewed and approved by the Ethical Review Sub-Committee Board for Human Research Involving Sciences, Thammasat University, No.3 (COA No.110/2561). The patients/participants provided their written informed consent to participate in this study.

## Author Contributions

NC, TS, KP, KY, PN, JL, and PK contributed substantially to the literature search and study design. NC, KP, KY, PN, and JL provided data collection. NC contributed to the data analysis, interpretation, and manuscript preparation. All authors critically reviewed the manuscript.

## Funding

The financial support provided by Thammasat University Research Fund under TU Research Scholar, Contract No.2/8/2562.

## Conflict of Interest

The authors declare that the research was conducted in the absence of any commercial or financial relationships that could be construed as a potential conflict of interest.

## Publisher's Note

All claims expressed in this article are solely those of the authors and do not necessarily represent those of their affiliated organizations, or those of the publisher, the editors and the reviewers. Any product that may be evaluated in this article, or claim that may be made by its manufacturer, is not guaranteed or endorsed by the publisher.

## References

[1] World Health Organization. World Health Organization (WHO) Website. (2020). Available online at: http://www.who.int (accessed January 26, 2019).

[2] From the global strategy for the diagnosis management and prevention of COPD Global Initiative for chronic obstructive lung disease (GOLD) (2020). Available online at: http://goldcopd.org

[3] Spruit MA Singh SJ Garvey C Zuwallack R Nici L Rochester C. An official American Thoracic Society/European Respiratory Society statement: key concepts and advances in pulmonary rehabilitation. Am J Respir Crit Care Med. (2013) 188:e13–64. 10.1164/rccm.201309-1634ST 24127811

[4] Spruit MA Nici L. Current concepts and definitions. In: Clini E editor. Textbook of Pulmonary Rehabilitation. Cham: Springer (2018). p. 19–22.

[5] Wouters EF Posthuma R Koopman M Liu WY Sillen MJ Hajian B. An update on pulmonary rehabilitation techniques for patients with chronic obstructive pulmonary disease. Expert Rev Respir Med. (2020) 14:149–61. 10.1080/17476348.2020.1700796 31931636

[6] Wadell K. Water-based exercise is more effective than land-based exercise in people with COPD physical comorbidities: critically appraised papers. J Physiotherapy. (2014) 60:57. 10.1016/j.jphys.2013.12.011 24856944

[7] McNamara RJ McKeough ZJ McKenzie DK Alison JA. Water-based exercise training for chronic obstructive pulmonary disease. Cochrane Database Syst Rev. (2013) 12:CD008290. 10.1002/14651858.CD008290.pub2 24353107 PMC12075999

[8] McNamara RJ McKeough ZJ McKenzie DK Alison JA. Water-based exercise in COPD with physical comorbidities: a randomized controlled trial. Eur Respir J. (2013) 41:1284–91. 10.1183/09031936.00034312 22997217

[9] Lotshaw AM Thompson M Sadowsky HS Hart MK Millard MW. Quality of life and physical performance in land- and water-based pulmonary rehabilitation. J Cardiopulm Rehabil Prev. (2007) 27:247–51. 10.1097/01.HCR.0000281772.28394.30 17667023

[10] Doig GS Simpson F. Randomization and allocation concealment: a practical guide for researchers. J.Crit Care. (2005) 20:187–91. 10.1016/j.jcrc.2005.04.005 16139163

[11] Miller MR Hankinson J Brusasco V Burgos F Casaburi R Coates A. Standardisation of spirometry. Eur Respir J. (2005) 26:319–38. 10.1183/09031936.05.00034805 16055882

[12] Black LF Hyatt RE. Maximal respiratory pressures: normal values and relationship to age and sex. Am Rev Respir Dis. (1969) 99:696–702. 5772056 10.1164/arrd.1969.99.5.696

[13] Mathiowetz V Dove M Kashman N Rogers S. Grip and pinch strength: normative data for adults. Arch Phys Med Rehabil. (1985)66:69–72. 3970660

[14] Jones SE Kon SS Canavan JL Patel MS Clark AL Nolan CM. The five-repetition sit-to-stand test as a functional outcome measure in COPD. Thorax. (2013) 68:1015–20. 10.1136/thoraxjnl-2013-203576 23783372

[15] Holland AE Spruit MA Troosters T Puhan MA Pepin V Saey D. An official European Respiratory Society/American Thoracic Society technical standard: field walking tests in chronic respiratory disease. Eur Respir J. (2014) 44:1428–46. 10.1183/09031936.00150314 25359355

[16] Singh SJ Puhan MA Andrianopoulos V Hernandes NA Mitchell KE Hill CJ. An official systematic review of the European Respiratory Society/American Thoracic Society: measurement properties of field walking tests in chronic respiratory disease. Eur Respir J. (2014) 44:1447–78. 10.1183/09031936.00150414 25359356

[17] Felcar JM Probst VS de Carvalho DR Merli MF Mesquita R Vidotto LS. Effects of exercise training in water and on land in patients with COPD: a randomised clinical trial. Physiotherapy. (2018) 104:408–16. 10.1016/j.physio.2017.10.009 30477678

[18] Becker BE. Aquatic therapy: scientific foundations and clinical rehabilitation applications. PM R. (2009) 1:859–72. 10.1016/j.pmrj.2009.05.017 19769921

[19] Wu W Liu X Liu J Li P Wang Z. Effectiveness of water-based Liuzijue exercise on respiratory muscle strength and peripheral skeletal muscle function in patients with COPD. Int J Chron Obstruct Pulmon Dis. (2018) 13:1713–26. 10.2147/COPD.S165593 29872289 PMC5973471

